# Understanding the perceived logic of care by vaccine-hesitant and vaccine-refusing parents: A qualitative study in Australia

**DOI:** 10.1371/journal.pone.0185955

**Published:** 2017-10-12

**Authors:** Paul R. Ward, Katie Attwell, Samantha B. Meyer, Philippa Rokkas, Julie Leask

**Affiliations:** 1 College of Medicine and Public Health, Flinders University, Adelaide, South Australia, Australia; 2 Political Science and International Relations, University of Western Australia, Perth, Western Australia, Australia; 3 School of Public Health and Health Systems, University of Waterloo, Waterloo, Canada; 4 Discipline of Paediatrics, Adelaide University, Adelaide, South Australia, Australia; 5 Sydney Nursing School, University of Sydney, Sydney, New South Wales, Australia; The Chinese University of Hong Kong, HONG KONG

## Abstract

In terms of public health, childhood vaccination programs have benefits that far outweigh risks. However, some parents decide not to vaccinate their children. This paper explores the ways in which such parents talked about the perceived risks and benefits incurred by vaccinating (or not vaccinating) their children. Between 2013–2016 we undertook 29 in-depth interviews with non-vaccinating and/or ‘vaccine hesitant’ parents in Australia. Interviews were conducted in an open and non-judgmental manner, akin to empathic neutrality. Interviews focused on parents talking about the factors that shaped their decisions not to (or partially) vaccinate their children. All interviews were transcribed and analysed using both inductive and deductive processes. The main themes focus on parental perceptions of: 1. their capacity to reason; 2. their rejection of Western medical epistemology; and 3. their participation in labour intensive parenting practices (which we term salutogenic parenting). Parents engaged in an ongoing search for information about how best to parent their children (capacity to reason), which for many led to questioning/distrust of traditional scientific knowledge (rejection of Western medical epistemology). Salutogenic parenting spontaneously arose in interviews, whereby parents practised health promoting activities which they saw as boosting the natural immunity of their children and protecting them from illness (reducing or negating the perceived need for vaccinations). Salutogenic parenting practices included breastfeeding, eating organic and/or home-grown food, cooking from scratch to reduce preservative consumption and reducing exposure to toxins. We interpret our data as a ‘logic of care’, which is seen by parents as internally consistent, logically inter-related and inter-dependent. Whilst not necessarily sharing the parents’ reasoning, we argue that an understanding of their attitudes towards health and well-being is imperative for any efforts to engage with their vaccine refusal at a policy level.

## Introduction

A number of assumptions underpin the prevailing mainstream discourses about parents in high-income countries who reject or are hesitant about vaccines for their children. Since the scientific and policy consensus on the benefits of nationally recommended vaccination schedules is unequivocal, vaccine delayers and refusers have been framed (particularly within lay discourse) as lacking knowledge, possessing ignorance, or being paranoid conspiracy theorists [1, 2]. The term ‘anti vaccination’ is often applied to such parents, which can serve to polarise or punish them for not complying with government policy [3]. Additionally, popular wisdom has long linked vaccine refusal to alternative lifestyles, and recent scholarship has attempted to synthesise evidence and advance explanations for how alternative lifestyles connect to vaccine refusal [4–8]. Overshadowing parental lifestyle decisions and practices is the changing framing of parenting, and expectations and moral obligations to be a ‘good parent’. In neo-liberal culture, parents are required to understand, make decisions and live life in terms of personal choice, “through the right to and problem of choice in the face of risk and indeterminacy” [9]. From a perspective of neo-liberal parenting, a failure to look after the self and children is characterised as a moral transgression [9]. Sensitivities around parenting have possibly increased as a consequence of the expansion of the ‘do it yourself’ guides to the parenting market. Within this context, a number of scholars have warned about the problems of blaming people for not vaccinating [6, 9, 10], and the need to understand parents’ ‘moral compass’ [11].

To address vaccine hesitancy and refusal in a constructive way, it is vital to understand the perspectives of those who do not vaccinate. Within this paper, we build upon recent work by Sobo [12] that reframes vaccine refusal from being a negative / deficit to, from these parent’s perspective, being a positively constituted act. Sobo identified (with her US Waldorf [Steiner] school vaccine refusing parents) that rejecting was also a certain type of *accepting*–accepting the predominant values, identities and lifestyles of one’s peers. Within this paper, we look in more detail at how parents view their hesitancy or rejection as informing and consistent with: 1. their capacity to reason; 2. their rejection of Western medical epistemology; and 3. their participation in labour intensive parenting practices (which we term *salutogenic parenting*), which they perceive as enhancing child health and preventing disease.

A conceptual framework for this paper draws on the concept of salutogenesis, originally coined by Antonovsky [13] to refer to what he saw as the origins of health. Relating to health promotion, Antonovsky [14] describes how conceptions of the body as ‘naturally’ healthy are flawed. He instead sees “the human system… as *inherently* flawed, subject to unavoidable entropic processes’ (p.13) and consequently seeks to move people along a continuum from ‘dis-ease’ towards health via ‘salutary factors’ “which are negentropic [and] actively promote health” (p.14). Salutogenesis, then, requires active participation so that the human body can remain at the optimum end of the continuum. We use the concept of salutogenesis in this paper to understand the parenting practices of our participants, which we term *salutogenic parenting*.*—*By salutogenic parenting, we mean engaging in practices that are believed by the parents to equip the child’s immune system with general health and resilience. Antonovsky’s orientation is away from ‘medical specialization’ and specific diseases framed as risk factors, and instead focuses on the general robustness of the human spirit and body. On this basis, even though a public health perspective regards vaccination itself as ‘health promoting’ and ‘illness preventing’, for some, vaccines are not salutogenic in that their use responds to specific risks with technological interventions. We consider that they, like Antonovsky, see the human body as an organism requiring conscious action and vigilance to maintain its health. Although a strong adherence to the idea of being ‘natural’ emerges, for them it is not akin to ‘leaving nature to its own devices.’ On the contrary, as we will show, salutogenic parenting involves a purposeful effort and intervention.

Given the challenges to public health that vaccine hesitancy or rejection poses, researchers are often eager to propose, trial and evaluate strategies for addressing and stemming it. Consideration of vaccine hesitancy or rejection in the context of salutogenic parenting will inevitably turn to addressing vaccine attitudes from within the parents’ own lifeworld and values. The evidence to date has shown it is very difficult to change behaviour among parents who actively refuse vaccination [15]. Providing the ‘correct’ information on vaccines improves knowledge but does not improve intent to vaccinate, indicating that simply ‘correcting myths’ about vaccines in information campaigns or public health interventions may not be effective in changing vaccination behaviours [2, 16–18]. In addition, trying to persuade non-vaccinating parents to accept vaccines is usually ineffective and often dissatisfying to both parents and health professionals [19, 20]. If Kata [21] is correct in stating that the views of vaccine rejecting parents are based on a post-modern philosophy that values a variety of forms of evidence and rejects the binary position of truth/fact emanating from the popular framing of science, then it would be near-impossible to change behaviours or beliefs emanating from science, medicine or the Government, since these are all sites of distrust [22]. Therefore, a key aim of this paper is to engage deeply with vaccine hesitant or rejecting parents’ worldviews, praxis and how (non)vaccination makes sense to them. We believe that our paper is a necessary starting point to understand the logic of non or selective vaccination from the perspective of parents who either do not, or selectively vaccinate their children, and one from which we believe more respectful and effective interventions can flow. As our paper engages with vaccine hesitant or refusing parents’ epistemology and lived experience, as socially situated individuals, we follow the lead of others [4, 7, 9, 10, 21] in arguing that only by understanding individual parents’ perspectives can policymakers truly engage with the implications of their hesitancy or refusal, and formulate strategies to address it. In this paper, we undertake an assets-based, as opposed to deficits-based, analysis of interviews with parents who either have rejected vaccinations or have selectively vaccinated their children. In so doing, this paper seeks to shed a light on the narratives of parents, from their perspectives, as opposed to advocating for or against their vaccination decisions. This process of ‘empathic neutrality’ [23] is discussed further in the following section of the paper. The paper focuses on the various parenting practices that the parents see as creating and maintaining health and wellbeing in their children, which they use to rationalise the lack of need for vaccinations.

## Methods

Although a detailed description of the methods and analysis has been provided in previous publication [22], we present a thorough description here for the reader. The previous paper focused on a different analysis of the data (mis-trust of pharmaceutical companies and other ‘expert systems’) and does not include any of the same quotes as the current paper.

### Ethics statement

Ethical approval for the study was obtained from the Flinders University Social and Behavioural Research Ethics Committee and The University of Western Australia (project number 6976 and permit RA 4/1/5890 respectively). Written informed consent was obtained from the study participants prior to commencement of the interviews.

Parents who had not vaccinated, partially vaccinated or delayed some vaccinations for their children were recruited from separate studies in Fremantle, Western Australia and Adelaide, South Australia. The focus of both studies was to understand parental decisions regarding childhood vaccinations, so we only recruited parents who had made an explicit, personal choice as opposed to the child’s under-vaccinated status being due to practical or logistical difficulties in accessing health services. Both studies used an interpretative, qualitative, methodological approach; undertaking semi-structured interviews to better understand parental vaccine hesitancy and influences upon parental experiences. Adelaide interviews (October-December 2015) targeted parents whose postal code was identified as having low immunisation coverage rates. Parents were recruited at a suburban organic community market and by snowballing. Fremantle parents (September 2013-April 2014) came from postcodes surrounding the City of Fremantle, which also had low immunisation coverage rates, and were recruited through posters, advertisements in the local newspapers, social media and snowballing.

Interviews in both studies focused on exploring the various factors that led parents not to (or selectively) vaccinate their children, including their broad perceptions of different vaccinations and vaccine preventable diseases. All interviews explored parents’ perceptions of different vaccine related information sources, including social media, health professionals and family/friends. Interviews also allowed the time/space for parents to talk about the various parenting practices they had developed, which is the primary focus of this paper.

The interviews did not set out in either study to probe parenting practices, but they emerged spontaneously as parents talked at length about how they attempted to make and keep their children healthy. We were very keen for the interviews to be open and non-judgemental, a process the Patton [23] called ‘empathic neutrality’. In discussing empathic neutrality as a key methodological criteria for good qualitative research, Patton [23] said that it allows the researcher to occupy “the middle ground between becoming too involved, which can cloud judgment and remaining too distant, which can reduce understanding” (p. 50). In order to operationalise empathic neutrality within the interviews, the researchers attempted to understand (not prove pre-existing) reasons for deciding not to (or to partially) vaccinate children. There was a non-judgemental stance towards whatever content emerged during the interviews and within our analytical and publication processes. We firmly believe that, in SA in particular, our empathic neutrality facilitated trust with our participants and led to positive feedback loops within their social networks, facilitating positive responses from subsequent participants. Since we used some snowball sampling, participants were very willing to talk with other non-vaccinating parents in order for their voices also to be heard, based (we believe) on our positive and non-judgemental interviews with them.

In total, 29 parents were interviewed, 9 from Fremantle (interviewed by KA), 20 from Adelaide (interviewed by PR). The majority of the participants were women (n = 25). The age range of parents was 25–50 years, 19 parents were aged between 36 and 42. The Fremantle parents were younger due to the age requirements of the youngest child. Over half of the parents held a university qualification. Thirteen participants had never vaccinated, 5 had ceased vaccinating, 7 were delaying or partially vaccinating, and 4 had previously delayed but were now up-to-date.

The interviews were transcribed verbatim and coded and analysed using the NVivo 10 software package. Three stages of analysis were undertaken: coding, conceptual categorisation and theoretical categorisation [24]. When the data were coded, words, or sections of text were ‘named’ using the actual words used by participants or by grouping similar words conceptually. Coding of a selection of transcripts was undertaken separately by PW, KA, JL, and PR in order to develop a shared and agreed coding framework. Conceptual categorisation was undertaken by grouping the initial codes into larger categories. This process involved an iterative process of inserting each of the initial codes into larger categories, based on their ‘semantic fit’ or the ways in which they seemed to be relating to a similar idea or issue. We examined the focused codes with respect to the theoretical and empirical literature on the sociology of parenting, alternative lifestyle or natural health practices, and codes were developed to group interview responses into themes relating to both praxis and values / opinions. This process highlighted data that both conformed to current theories and ‘new insights’. In particular, the researchers explored differences and similarities between participants with different histories of vaccinating their children, some of whom originally vaccinated but ceased, some who delayed but eventually caught up, and some of whom have never vaccinated their children. Frequent discussions within the research team validated emerging codes. All names have been replaced by pseudonyms.

## Results and discussion

The parents in this study offered three general and interconnected rationales for their rejection or delay of some or all vaccines for their children: “capacity”—their perception of their own cognition and abilities to make and keep their child healthy; “knowledge”—their specific engagement with, and trajectory through, the scientific evidence and government recommendations; and “salutogenic parenting”—their comprehensive preventative and health promoting practices which, they believed, replaced the need for vaccines. Capacity, epistemological critiques of medical and scientific knowledge and specific salutogenic activities mutually and repeatedly reinforced each other, such that parents saw their rejection, selection or refusal of vaccines not as a deficit, but rather as an asset.

### Capacity: A conscious and logical choice not to vaccinate

The *choice* to vaccinate or not was a central theme running through all interviews, based on a process of gathering information and making a conscious decision. This fits with the notion of the ‘freedom of the subject’ within neoliberal parenting [9]. Parents, first and foremost, saw themselves as active and capable agents who could (and should) arm themselves with information and act in the best interests of their children. They believed that their capacity qualified them sufficiently to make good decisions, and were quick to point this out, particularly in the face of those who thought otherwise. Pippa (SA) felt that her research and informed choice not to vaccinate her child was not met with the respect she felt she deserved,

I’m reading some stuff that leads me to question, and then each interaction I’ve had with medical professionals leaves me feeling very disenchanted, patronised or outright lied to. I was so shocked by that. At that point I had finished my Master’s degree. I mean, I’m not an idiot.

Several participants had changed their views about vaccinations over time, originally vaccinating their child but then, in their words, ‘doing more research’ and ‘becoming more educated’. This constant quest for research and education fits with the work of others in the US [4, 6, 25] and, for our participants, the increased effort in research generally resulted in the decision to cease vaccinating. For example, Kavita (SA), who started out vaccinating her son, said,

I just did it because I thought I was meant to, and it really doesn’t sit well with me anymore.

The parents’ perceptions of their own capacity–which they sometimes explicitly recounted as developing over time–were key to their belief that they were fully capable of making informed and reasoned decisions. This capacity differentiated them–in their view–from other parents who did not vaccinate for reasons that have been identified as being linked to system failure and social disadvantage [26]. The parents in our study literally perceived their own unvaccinated children (on the basis of research and choice) as being qualitatively different from other unvaccinated children (on the basis of ‘going along with the herd’). For example, Charlotte saw no problem with vaccine refusal occurring on a larger scale, so long as it was accompanied by appropriate reasoning and buttressed by suitable behaviours.

*I think if there were more people unvaccinated*, as long as they were doing it consciously *and–and*, *like*, *it’s not just about alternatives*. *It’s about creating a good energy in your life*, *creating good energy with your relations*, *with your work and giving yourself good food which comes from the earth*, *not from a packet*. *All of those things contribute to health; it’s not just about vaccinating*.

Already, readers will note participants hailing the specific lifestyle activities that we will document later in the paper. On this basis, we state now (and will reiterate) that the rationales informing our parents’ refusal of vaccines are *not* chronological, but rather circular and mutually reinforcing. The parents’ lifestyle praxis may indeed precede their sense of capacity, rather than follow it as the structure of our article might suggest. Hence, our job is not to show how any one of these factors cause the others, but rather how they become part of a self-referential logic of care for parents.

### Questioning science/shifting evidence

Parents who self-consciously value their capacity to reason also value their capacity to question and distrust traditional scientific evidence, and formulate alternative systems of parsing evidence. Similar to previous qualitative studies in the US [6, 9], parents in our study talked at length about not only the evidence underpinning vaccinations, but more broadly about the evidence underpinning numerous health-promoting and illness-preventing activities they undertook with and for their children. Their interrogation of, and engagement with, this evidence took the form of critiquing Western medical epistemology and making space for a different one. Consequently, much of what they had to say related to the very nature of knowledge and evidence itself. Kaufman [9] argues that parents become ‘bricoleurs’ who put together ‘fragments’ of information, trust, rumour, folklore and partial expertise in order to be ‘subjects of freedom’ and also good parents. Parents then, “use those fragments first, to explain to themselves the medicocultural phenomenon in which they find themselves embedded, and second, to act responsibly on behalf of their children” (p. 27). This certainly resonates with our study. For example, Kavita (SA) talked about the different knowledges around vaccinations, stating that people need to gather their own evidence before blindly trusting a doctor,

*If I had a friend now who was pregnant and came to me and said “I’ve been recommended the flu vax; should I have it?’ I would say ‘in my opinion, no, but do your research. Before you just trust in that doctor, just do your research and make your informed decision. Your doctor is saying that to you based on his knowledge base and that might end up being quite different to your knowledge base*.

Despite their sense of their own capacity, the different forms of evidence they utilised made it difficult for a number of participants to make ‘informed choices’ around vaccinations. Meg (WA), for example, rejected homeopathy as not evidence-based, and was therefore ‘stuck.’ Katie (SA), meanwhile, along with many participants, questioned the scientific validity of herd immunity, and therefore was not prepared for her child to be at potential risk from side-effects of the vaccination, *‘Why should my child take one for the team for herd immunity*, *which is a theory that’s never been tested amongst the vaccinated*?*’*. Similar questioning of herd immunity by vaccine rejecting parents has been found elsewhere [27]. Owen (SA) talked about the difficulties involved in searching for and synthesising evidence in order to make the best possible decisions for his children, and how he is open to new information,

to me it isn’t a closed door… it’s an enquiry about a lot of things, but that’s kind of my life; it like takes up a lot of RAM, all this questioning… I’m grasping with it, you know, to make the best decision because all of my things are about making the best decision. You know, about the vaccinations… I haven’t been able to let go totally of it and say ‘oh fuck that’. There’s still a question… it’s bloody challenging. I know it’s a done deal for my wife but I’m not a closed—you know, I’m still wondering.

Although there was some uncertainty and even anxiety caused by attempts to synthesise many and varied forms of information from different sources, it has been argued that “calls to ‘do your own research before vaccinating’ dovetail with the postmodern characteristics of patient empowerment and shared decision-making, where individuals play a more involved role in their healthcare” [10] (p. 3784). However, Kata argues that in what she terms the Postmodern Pandora’s Box, ‘facts’ are simply seen as another form of ‘opinion’, which can be agreed with or disagreed with–all knowledge is corrigible. She goes on to say that “this issue is as much about the cultural context surrounding healthcare, perceptions of risk, and trust in expertise, as it is about vaccines themselves” (p. 3784). In this way, parental decisions about childhood vaccination may be best understood at the level of epistemology–understanding the epistemological landscape of parents and engaging with them at that level–rather than honing our gaze to the scientific evidence on risks/benefits of the vaccine (and trying to change behaviours by proving more information on risks and benefits of vaccinating children) [10].

Our findings about the anxiety created by ‘reading widely’ and considering multiple forms of evidence in the Postmodern Pandora’s box are slightly at odds with the parents interviewed by Sobo et al [25] in the US. Like us, Sobo and colleagues found that the ‘reading widely’ process involved lots of research and gave parents pride in themselves and a sense of responsibility and reasoned justification for their decisions. However, Sobo et al argue that parents “valorized multivocality” (p. 539), whereas some of our participants felt that the multi-vocality led to further questioning and a never-ending quest for the ‘right’ answer. We perceive their search for truth as a (perhaps futile) realist quest in a postmodern paradigm; our parents displayed an ontological need for closing down rather than opening up possibilities. Whilst they recognised various forms of knowledge and often questioned ‘expert’ knowledge in the form of science and allopathic medicine, it seems that they were also trying to reduce the ontological insecurity inherent in Postmodern Pandora’s Box.

### Salutogenic parenting—comprehensive health promoting and illness preventing activities

The third feature of the parents’ rationale for refusing vaccines was the most comprehensive: salutogenic parenting. Our parents’ heightened sense of capacity and rejection of mainstream Western epistemology corresponded to an identity and praxis so totalising that it came to define their identity and sense of self. Participants talked at length about the variety of health promoting and illness-preventing activities they engaged in, within which *not vaccinating* their children was contextualised and justified. Their praxis, eclectic yet often uniform, was held together by a milieu and identity that appeared utterly coherent to those within it. Parents’ activities included managing nutritional intake during pregnancy, breastfeeding, feeding their children organic and/or home-grown food, cooking from scratch to reduce preservative consumption, reducing their children’s exposure to chemicals and toxins and promoting physical activity and play-based learning. There were also a lot of statements about ‘withdrawing’ from or being ‘unplugged’ from mainstream society and wanting to live ‘off the grid’, including not watching commercial TV or listening to commercial radio, not buying products from supermarkets. Often, this had been a long time in the making. Holly (SA) talked about the development of her logic of care over time, from before her child was born.

I just found that for me it just didn’t sit right to go ahead with it, and that I needed to really look at the health of my child overall, the lifestyle that we were living and the conditions that we live in and look at strengthening her immune system overall rather than looking for a vaccination.

Dianne (SA) talked about the variety of activities and practices that she engaged with, for the physical, emotional and psychological health of her daughter,

I stepped back… I think it was great for me because it enabled me to achieve a certain lifestyle, creating my own food from scratch. I grow vegetables and I have chickens I mean, I feed my children organic food, I cook everything from scratch. I don’t give them processed food. We have no chemicals in the house. We don’t drink fluoridated water, we drink rainwater which has been filtered. So why would I then go and put all those chemicals in my child?

Dianne linked the lack of chemicals in her daughter’s food with her perception of chemicals in vaccinations, thereby drawing a logical chemical-free link to not vaccinating her child. She went on to talk about her reasons for limiting TV access to her daughter,

I have a TV but we don’t watch it very often. They are limited to one channel. I don’t like them watching adverts because of the commercialism. I think it’s stuffed down their throats all the time and I think it certainly affects children as they’re growing up.

There were numerous examples from participants of ‘bad chemicals’ and pollutants in food, clothes, mattresses, sunscreens, paints, chairs and the endemic problem of weedkillers (glycophosphate) being ‘sprayed everywhere’, which was seen as toxic and carcinogenic. ‘Bad chemicals’ were primarily avoided in food (hence organic where possible) and in cleaning products in the home. In the SA participants, where attitudes towards health promoting practices were probed in more detail, all participants talked at length about the benefits of organic food, which was linked to the fruit and vegetables having no chemicals. Some of the first participants in SA were originally recruited from an organic market in Adelaide, which may have skewed some of the discussion during interviews, although later participants were recruited through snowball sampling and they also talked at length about organic food. Holly (SA) talked about her strategies for both trying to prevent and treat illness through healthy eating. In attempting to prevent illness, Holly said,

… I look at the whole picture of the organism, I guess. So my children eat the best quality food, whole food. Eat a rainbow, as I would say, in colour. Our water is—we’ve got a lovely filter on the outside of our house so we’ve got nice, fresh, clean water coming through. We get sunshine every day. Play outside every day. We eat organic.

Kavita described her use of coconut oil as a sunscreen to avoid the chemicals in commercial sunscreen,

It’s got a natural SPF in it so that’s what we generally use. If we were going to be out in the sun all day I would use a more natural version of sunscreen or I’d just put a long sleeved top on him and a hat… I’m probably more frightened of the chemicals in sunscreen than I am of good doses of sun.

However, products marketed as ‘natural’ were also questioned by some participants, who undertook research on ingredients in order to feel comfortable in giving them to their children. This demonstrates their endless cycle of vigilance, their sense of capacity to question, research and make their own decisions, and their mistrust of forces that might seek to exploit them. Dianne (SA) said

I’ve got a lot of mistrust so I think just from—you know over the last few years, going down this alternative route, realising that all the packets in the supermarket which say ‘natural’ on them doesn’t mean anything, that when you actually start to research and look at the ingredients, especially GMO and Monsanto, that’s something I’ve got quite passionate about.

In SA, a number of participants sent their children to independent schools, such as Steiner and Montessori, due to the perceived weaknesses of public schools for dealing with individual learning needs and the benefits of the philosophies that underpin these particular pedagogical methods. Similar schooling choices have been found in the US with non-vaccinating parents [5, 6]. Charlotte (SA) talked about how her interactions with her children, her limits on TV, her emphasis on outdoor play and natural materials fitted with those of Steiner schools,

That’s a thing that comes with the whole Steiner philosophy, that TV really isn’t good for them. Their brain is developing and growing and TV is just like messing with that… they rarely get to touch my phone. …they come home and they play they read, they play outside; they get out in nature. Like, it’s the type of lifestyle that we’ve built for them…most of their toys are wooden or made from natural fibres like wool or fleece. It’s really encouraging them to play and to create their own kind of play.

Charlotte’s description of Steiner philosophy gelled with her own broader logic of care. Charlotte grew her own vegetables, bought organic food, cooked from scratch, did not use chemicals, did not trust government or big business (rarely shopped in supermarkets) and did not vaccinate her children. She regarded all these practices as logical and internally consistent in promoting the health of her family–salutogenic parenting.

In addition to the comprehensive explanations of the logic of care already outlined, participants talked specifically about breastfeeding and homebirths as health promoting and illness preventing activities for their children, improving natural immunity and therefore, as they saw it, negating the need for vaccinations. Wu et al [28] found that women who intended to breastfeed were less likely to trust childhood vaccinations in one US hospital study. Sonya (SA) talked about the benefits of breastfeeding and homebirth for her daughter, while Kavita breastfed her son for 28 months as a way of developing natural immunity to illness. Katie also talked at length about the importance of building up natural immunity in children as a form of protection, and in particular what she saw as the importance of breastfeeding in this process,

the fact that they even link the word ‘immunity’ with vaccinations is offensive. It’s like the only way to get immunity is to actually have the disease and have your body build up the natural immunity to it. … [T]hat is the normal way that we all survive, you know. Our mothers built up their own natural immunity, then the babies come and they receive their mum’s immunity for the first couple of years while they’re being breastfed so they’re being protected there. But when you’ve got all these [vaccinated] mums coming through that don’t have the natural built up immunity, you know, all they have is all these old vaccines that no longer work and have dampened their body’s ability to defend itself, so those kids are not getting that immunity from … their mum…

Our findings in relation to salutogenic parenting are relevant to other analyses of the sociology of parenting [29]. Concepts of ‘intensive mothering’ [30] and ‘concerted cultivation’ [31] have become a cultural logic for middle-class mothers. In this way, women with the requisite cultural and material resources are morally urged to spend increasing amounts of time, energy and money in raising their children. This reflects our sample of parents, who were generally middle-class and could financially afford things like organic foods, private education, non-traditional toys and complementary and alternative medicine. Applying this purely economic lens, which our participants did not use, one could say that salutogenic parenting has added resource implications. Not only does salutogenic praxis cost more money, it also requires additional time, energy, social and cultural capital–whether that be finding good places to purchase organic food, commuting to the closest Montessori school, or time and/or networks to source naturopaths (see additional reference for a more comprehensive discussion of resource implications [8]).

The mothers interviewed by Sobo also talked about the similar lengths they went to in order to perform ‘intensive parenting’ [5, 6, 25]. Lois [32] shows how mothers who home-school consider that they are putting their children at the centre of their lives, and Avishai [33] shows how mothers who breastfeed see that as a further commitment to the health of their children. As a sociological critique, Reich argues that when women make vaccine-related decisions for their children, “they do so as educated, partnered and privileged women” [4] (p. 683)–this resonates with most of the female respondents in our study. Reich [4] argues that “women see themselves as experts and aim to be great mothers by stressing their individual choices to optimise their own children’s health and wellbeing” (p. 683). However, rather than simply rationalising or describing what Jenny McCarthy called “mommy intuition” [4] (p. 683), we need to situate mothers within their social, cultural and economic context. “[I]t is difficult to distinguish a ‘mother’s intuition’ from ideas arising from a woman’s social role, a woman’s upbringing, and the culture of motherhood” [30] (p. 72). Amongst Reich’s subjects, one of the key findings was that “in touting the superiority of homemade baby foods, breast milk, or organic products, mothers confidently communicate their ability to protect against illness, reiterate their sense of expertise and control, and highlight their advocacy for their children as care work, all of which protect children from disease without expert intervention or vaccines” [4] (p. 694). This succinctly summarises the salutogenic parenting practices of the participants in our study.

## Concluding comments

Parental decisions and logic around non- (or selective) vaccination involved the inter-relation and reinforcing of ‘capacity to make decisions’, ‘questioning science’ and ‘salutogenic parenting’. On their own, these are not new within the extant literature, and have been identified as discrete ‘reasons for non-vaccination,’ particularly in US literature [4–7, 9, 25]. We believe that our distinct contribution to knowledge lies in highlighting the inter-weaving and self-referential nature of these properties. In the same way that Giddens refers to the ‘duality of structure’ as agency and structure requiring each other for their existence [34], our ‘trilogy of structure’ means that each of our identified themes requires the others for their ongoing existence and relevance for parents; together they define the logic of care underpinning vaccination decisions.

Our key finding around salutogenic parenting both dovetails with and deviates from recent research in the US [4–6, 12]. Reich [4] found that mothers trust their own judgement more than experts (drawing on embodied knowledge and intuition). They assessed risks of various diseases and saw some as lower than the risk of vaccination. This was similar to how our participants constructed their capacity to undertake their own research. However, Reich argued that vaccine refusing mothers “risk neither contributing to public health nor acknowledging the broader communities in which they live” (p. 697) and that the “neoliberal mothering project, which focuses on one’s own children, allows mothers to ignore how their unvaccinated children benefit from other children’s vaccinations and how their children might present a risk to others” (p. 697). Rather, our participants genuinely believed that they posed little/no risk to others because they perceived their children as sufficiently healthy, as a direct result of their salutogenic parenting. Their focus, whilst consistent with the concept of neoliberal mothering, was squarely placed on creating and maintaining healthy children (an assets-based approach) as opposed to seeing (but ignoring) that their children posed potential risks to others due to their non-vaccinated status (a deficits-based approach). As we have noted, they actually saw their children as qualitatively different from unvaccinated children who were not being raised on a salutogenic regimen.

Sobo et al’s [12] division of parents who questioned vaccinations into four quadrants is also pertinent to our analysis and findings. Their four quadrants related to a) whether parents saw allopathic medicine or natural remedies as valued methods for maintaining health, and b) whether they thought the world was full of risks and therefore individual responsibility is required, or whether the world is safer and they can therefore consider community benefits [25]. ‘High risk/allopathic’ parents vaccinated their children to protect them and their families from disease, whereas ‘low risk/allopathic’ parents vaccinated often without much reflection, because they regarded it as a social norm and the ‘right thing to do’ for others. The ‘high risk/natural’ parents had an individualistic approach to looking after their children, but did not see vaccinations as beneficial. The ‘low risk/natural’ parents did not have vaccinations on their radar, since they pursued a ‘natural lifestyle’ as an end in itself, rather than to help the body fight diseases. We conceptualise our parents as falling into both of these latter groups. Sobo’s ‘high risk/natural’ parents regarded their health promoting parenting behaviours as making more sense than vaccinating, included breastfeeding, reducing chemicals, eating good food, and we saw some of our parents engaging in these practices as a conscious negation of the need to vaccinate. We note the stress on high level inputs here that adheres to Antonovsky’s formulation of salutogenesis [14]. Left alone, entropy happens, so bodily health takes work and effort. However, many of our participants also fitted into the ‘low risk/natural’ category, wherein salutogenic parenting is both the means and an end. Sobo’s parents in this category–and some of ours–focused their attention on salutogenic parenting to maintain health and good living for their children, and as a result, they felt that they did not need to vaccinate–non-vaccination was almost a bi-product rather than a focus. Our ‘low risk’ parents’ approach at times appeared carefree, and yet the inputs still present in their parenting would not be at odds with Antonovsky either. Whether high risk or low risk, the parents were putting in the work of salutogenic parenting that, at its heart, was dedicated towards their children’s health and wellbeing.

Sobo et al also talked about ‘curated assemblages’ [25] (p. 537) which were collections of stories, memories and practices, all held together by “connections envisioned by the individual curator” (p. 537). Kaufman [9] used a similar concept of “bricolage". The (non)place of vaccinations in this assemblage was, for most of our parents in our study, the beginning, middle and end of the unfolding narrative. Sometimes, the decision not to vaccinate was part of a ‘low risk/natural’ standpoint, whereby vaccinations simply had no place in the parents’ lifeworld and unfolding narrative. However, participants fitting this description were less prevalent. It was more usual for parents to initially consider vaccinations and maybe consent to early vaccination (often with a first child), and then to reconsider vaccinations due to perceived adverse events for self or others. This reconsideration involved copious research and then seemed to go hand in hand with other life and lifestyle practices which can be constructed as ‘natural’. This is analogous to the ontological quandary “what came first, the chicken or the egg?” What came first, the natural lifestyle and practices (which lead to vaccine rejection) or vaccine rejection (which leads to a reconsideration of lifestyle principles and practices)? While our study was not designed to answer this question, we believe that the cyclical logic we have identified might bring us closer to understanding the interrelation of these two components, laying a foundation for future research. The answers may well help to work out the most ethically and socially appropriate forms of communication with vaccine rejecting parents. Sobo argues that we need to learn from parents and then provide communication in culturally consonant ways, such as emphasising how vaccines help a child’s immune system to ‘self-strengthen naturally’ [6] (p. 394), and how possible side-effects of vaccine-induced fever could be re-worded as indications of “self-strengthening and bodily renewal,” which “demonstrat[e] … that the benefits of childhood illnesses are not being foregone” (p. 394). In this study, we have learnt from our participants and conclude that reinforcing their self-conception as free-thinkers, good parents and knowledgable people would likely provide resonant messaging.

Whilst our paper has major strengths in terms of theoretical and empirical depth, we note some potential limitations that need to be borne in mind when attempting to infer from our findings. Firstly, we have analysed data from two different studies, undertaken by two different researchers in different Australian cities. Whilst the different studies aimed at understanding parental decision-making and rationalities, there may be some contextual differences which make our merging of data less than optimal. However, both of the researchers are authors on this paper (KA and PR), were involved in the analysis of the merged dataset and attest to the comparability of data. Secondly, whilst the parents in both studies had either partially or fully rejected vaccinations for their children, they do not necessarily represent the views of all parents who reject (partially or fully) vaccinations, although this is the case for all qualitative research whereby the epistemological basis is about generating understanding rather than explaining patterns.

Further research aimed at understanding if/how Sobo et al’s high- and low- risk ‘natural’ quadrants play out in different social and cultural contexts is required, since different forms of communication would be required for parents in the different quadrants. If parents perceive little/no risk about illness and disease (i.e “it’s the natural order of things to get illness and part of the universe”) and believe in natural forms of health promotion, then talking about the risks of measles or the benefits of preventing vaccine preventable diseases would be close to pointless. Similarly, if parents perceive high risks from diseases but believed in natural remedies and treatments, then talking about vaccines in purely scientific and medical terms may be ineffective, but talking about how vaccines can boost natural immunity might work better. Indeed, when messages about ‘evidence of safety’ are constantly *communicated to/at* non-vaccinating parents in the hope of changing their behaviours, they may in fact reinforce a position of non-vaccination because parents do not trust science to provide appropriate evidence [35].

One thing we do know is that doctors (in general) and pharmaceutical companies (en masse) are distrusted by vaccine rejecting parents [22, 28, 36, 37]. The central question for public health, therefore, is: if the messenger is distrusted, how do we get the message there in an appropriate manner and what message will be accepted? In order to communicate effectively, ethically and appropriately with parents, we need to understand ‘who they are’ and what they represent’. Useful here is Ward’s [4] suggestion that there are three main groups involved in vaccine resistance: 1) People against ALL vaccines in ALL forms, who establish boundaries to their social movement as a form of scientific and political legitimacy (‘anti-vaccine’); 2) people with particular political struggles focused on natural lifestyles, CAM, environmental concerns, which can include vaccine rejection/criticism, but often is focused on other issues; and 3) people with a set of political struggles, which under certain circumstances, may include vaccinations. These last two may be more the ‘vaccine hesitant’ as opposed to ‘vaccine rejecters’. We believe that the majority of our participants were a mix of the first two groups.

A number of researchers argue that policy and practice needs to focus on individuals whom Ward [3] would characterise as groups two and three, because they may be more open to change [1, 12, 21, 38, 39]. (We note, however, that our focus was on understanding parents’ logic for vaccination decisions, as opposed to explicitly seeking to change their behaviours). Interestingly, we found no discernible difference in parenting practices between (Ward’s category of) ‘anti-vaccine’ and parents and those whom we would class as more ‘vaccine hesitant.’ All the parents made a conscious and (for them) logical choice not to vaccinate, questioned the science underpinning vaccinations and undertook a myriad of salutogenic practices to promote the health and wellbeing of their children. This shows us how important the framing of researchers can be to what we observe; depending upon which lens one views such parents through, one obtains a very different picture. Placing vaccines at the centre of parents’ decisions could lead us to bifurcate them into ‘anti vaccination’ and ‘vaccine hesitant’. Indeed this categorisation may have utility in raising the popular discourse from the low based of the “pro” and “anti” vaccination dichotomy. However, analysing the processes and logic underpinning their parenting practices leads us instead to a unification, whereby all parents have come to similar decisions and are following similar paths of salutogenic parenting, irrespective of the ideological basis for their vaccination choices.

As researchers operating in the realm of public health, we do not wish to valorise the decisions of parents not to vaccinate their children. We fully believe in population-level vaccination programs, and have reflexively engaged with this position *vis a vis* that of our participants, through a process of empathic neutrality. However, we also recognise the great lengths that our participants go to in order to develop and maintain health and wellbeing (in an holistic sense) for their children. Our data show that attempts to pathologise ‘non vaccinating parents’ and construct them as non-compliant and risky for population health does not recognise their perceived logic and salutogenic parenting, and simply leads to further polarisation and distrust. However, purely focusing on the intensive and positive parenting practices, but recognising non-vaccination as being part of a ‘good parent’, ignores the potential risks incurred for them and for population health. Further research is required to develop ethically and socially appropriate conceptualisations of and communication strategies with such parents. These strategies would recognise their salutogenic parenting but also recognises the need, from a public health perspective, for increased childhood vaccination rates, thereby engendering two-way trust between non-vaccinating parents and relevant instruments of government.
